# Implementing interventions to promote spectacle wearing among children with refractive errors: A systematic review and meta-analysis

**DOI:** 10.3389/fpubh.2023.1053206

**Published:** 2023-03-10

**Authors:** Linrong Wu, Jiayi Feng, Mingzhi Zhang

**Affiliations:** ^1^Joint Shantou International Eye Center (JSIEC) of Shantou University & The Chinese University of Hong Kong, Shantou, China; ^2^School of Public Health, Shantou University, Shantou, China

**Keywords:** spectacle, refractive error, compliance, children, meta-analysis

## Abstract

**Purpose:**

To investigate the level of compliance of children with refractive errors who are provided free spectacles, and to identify the reasons for non-compliance.

**Methods:**

We systematically searched the PubMed, EMBASE, CINAHL, Web of Science, and Cochrane Library databases from the time these databases were established to April 2022, including studies published in English. The search terms were “randomized controlled trial” [Publication Type] OR “randomized” [Title/Abstract], OR “placebo” [Title/Abstract]) AND ((“Refractive Errors”[MeSH Terms] OR (“error refractive” [Title/Abstract] OR “errors refractive” [Title/Abstract] OR “refractive error” [Title/Abstract] OR “refractive disorders” [Title/Abstract] OR “disorder refractive” [Title/Abstract] OR “disorders refractive” [Title/Abstract] OR “refractive disorder” [Title/Abstract] OR “Ametropia” [Title/Abstract] OR “Ametropias” [Title/Abstract])) AND (“Eyeglasses” [MeSH Terms] OR (“Spectacles” [Title/Abstract] OR “Glasses”[Title/Abstract]) AND (“Adolescent” [MeSH Terms] OR (“Adolescents” [Title/Abstract] OR “Adolescence”[Title/Abstract]) OR “Child”[MeSH Terms] OR “Children”[Title/Abstract])). We only selected studies that were randomized controlled trials. Two researchers independently searched the databases, and 64 articles were retrieved after the initial screening. Two reviewers independently assessed the quality of the collected data.

**Results:**

Fourteen articles were eligible for inclusion, and 11 studies were included in the meta-analysis. The overall compliance with spectacle use was 53.11%. There was a statistically significant effect of free spectacles on compliance among children (OR = 2.45; 95% CI = 1.39–4.30). In the subgroup analysis, longer follow-up time was associated with significantly lower reported ORs (6–12 vs. <6 months, OR = 2.30 vs. 3.18). Most studies concluded that sociomorphic factors, RE severity, and other factors contributed to children not wearing glasses at the end of the follow-up.

**Conclusion:**

The combination of providing free spectacles along with educational interventions can lead to high levels of compliance among the study participants. Based on this study's findings, we recommend implementing policies that integrate the provision of free spectacles with educational interventions and other measures. In addition, a combination of additional health promotion strategies may be needed to improve the acceptability of refractive services and to encourage the consistent use of eyewear.

**Systematic review registration:**

https://www.crd.york.ac.uk/PROSPERO/display_record.php?RecordID=338507, identifier: CRD42022338507.

## 1. Introduction

In 2020, 157 million people worldwide were affected by uncorrected refractive errors, which have become the primary cause of moderate or severe vision impairment on a global scale (1). In particular, the prevalence of myopia and high myopia is predicted to affect five and one billion people, respectively, by 2025 (2). Refractive error is increasingly regarded as a serious global public health problem. Although it can be safely and affordably corrected using precise spectacles, a high proportion of children exhibited low compliance with spectacle wear. Therefore, it is imperative to implement interventions to increase the rate of compliance with spectacle wear among affected children.

A meta-analysis of the economic costs of the global burden of myopia showed that the potential loss of productivity due to uncorrected myopia is substantially greater than the cost of correcting myopia (3). Providing spectacles to all individuals with a significant refractive error has been recommended by the World Health Organization's VISION 2020 targets to control blindness in children (4). One study reported that the number of individuals wearing spectacles was two-fold higher in the group that received spectacles free of charge than in the group that did not receive free spectacles (5). However, recent studies found that receiving free spectacles did not effectively improve compliance among the study participants (6–10). Although cross-sectional studies were conducted to analyze the effect of free spectacles on children's compliance with spectacle wear and the overall compliance of spectacle use in a recent meta-analysis of data from 20 studies was considerably low at 40.14% (11) in 2018, to date, no RCT studies on the effect of providing free spectacles on children's compliance with spectacle use have been conducted. Given the stronger causal association of RCT studies, to better address the inconsistencies in the literature as well as to assess the effect of the provision of free spectacles on improving children's compliance, we conducted this systematic review and meta-analysis based on recently published randomized controlled trials. In addition, some of the reasons why children with refractive errors do not wear spectacles require further research owing to our observation that spectacle ownership among migrant children requiring eyeglasses in cities in eastern China may be lower than that of local children (12), and we conducted a more comprehensive analysis of the reasons.

## 2. Method

The study protocol was registered in PROSPERO, ID: CRD42022338507.

### 2.1. Search methods

Two authors searched PubMed, CINAHL, Cochrane Library, EMBASE, and Web of Science databases (from inception to 9 April 2022) using the MeSH search strategy, including subject headings and free text terms relevant to refraction error, spectacles, and children (or adolescents). The search terms were “randomized controlled trial” [Publication Type] OR “randomized” [Title/Abstract], OR “placebo” [Title/Abstract]) AND ((“Refractive Errors” [MeSH Terms] OR (“error refractive” [Title/Abstract] OR “errors refractive” [Title/Abstract] OR “refractive error” [Title/Abstract] OR “refractive disorders” [Title/Abstract] OR “disorder refractive” [Title/Abstract] OR “disorders refractive” [Title/Abstract] OR “refractive disorder” [Title/Abstract] OR “Ametropia” [Title/Abstract] OR “Ametropias” [Title/Abstract])) AND (“Eyeglasses” [MeSH Terms] OR (“Spectacles” Title/Abstract] OR “Glasses” [Title/Abstract]) AND (“Adolescent” [MeSH Terms] OR (“Adolescents” [Title/Abstract] OR “Adolescence” [Title/Abstract]) OR “Child”[MeSH Terms] OR “Children”[Title/Abstract])). We included studies that were published in English. We also performed forward citation tracking and reference list screening for eligible studies. The titles and abstracts of articles were screened by two authors based on the inclusion and exclusion criteria. Subsequently, we screened the full text and extracted data. Any disagreement was resolved by discussion until a consensus was reached or, if necessary, a third author was consulted.

### 2.2. Inclusion and exclusion criteria

The inclusion criteria were as follows: (a) randomized controlled trials designed for children with refractive errors including myopia, hypermetropia and astigmatism; (b) interventions to provide free spectacles; (c) age limit being children (i.e., individuals < 18 years old); and (d) outcome of self-reported or observed spectacle wearing.

The exclusion criteria were as follows: (a) review or meta-analysis, abstract, letter, comment, conference or case report; (b) republished literature; (c) research published in other language than English. (d) studies that had missing raw data or errors; and (e) studies that involved other interventions beyond free spectacles (such as the provision of custom glasses, contact lenses, and keratoplasty lenses).

### 2.3. Data extraction and analysis

Two authors extracted the following data: the first author's name, year of publication, country or area where the study was conducted, inclusion and exclusion criteria, sample size, age of the participants, intervention and control details, duration of follow-up outcomes assessed, and the number and percentage of the compliant and non-compliant participants. After the extraction process, all related data were entered into Microsoft Excel for compilation. Two reviewers conducted quality assessments independently. We assessed publication bias using Egger's test and asymmetric funnel plots and then used Trim-and-Fill analysis to adjust the OR (95% CI) to determine if publication bias existed. Sensitivity analyses were also performed to calculate pooled estimates for the remaining studies by removing one study at a time to determine whether the result depended on a particular study. Heterogeneity tests and statistical analyses were performed using Revman 5.3. A forest plot was used to generate a pooled compliance estimate for children's spectacle use. A subgroup analysis was conducted, which distinguished between studies based on the duration of the follow-up and the mean age of the participants.

### 2.4. Synthesis

We conducted a meta-analysis for outcomes where more than one study assessed the same outcome for a similar intervention and narrative syntheses of other outcomes and studies.

## 3. Results

### 3.1. Study characteristics

**Figure 1:**
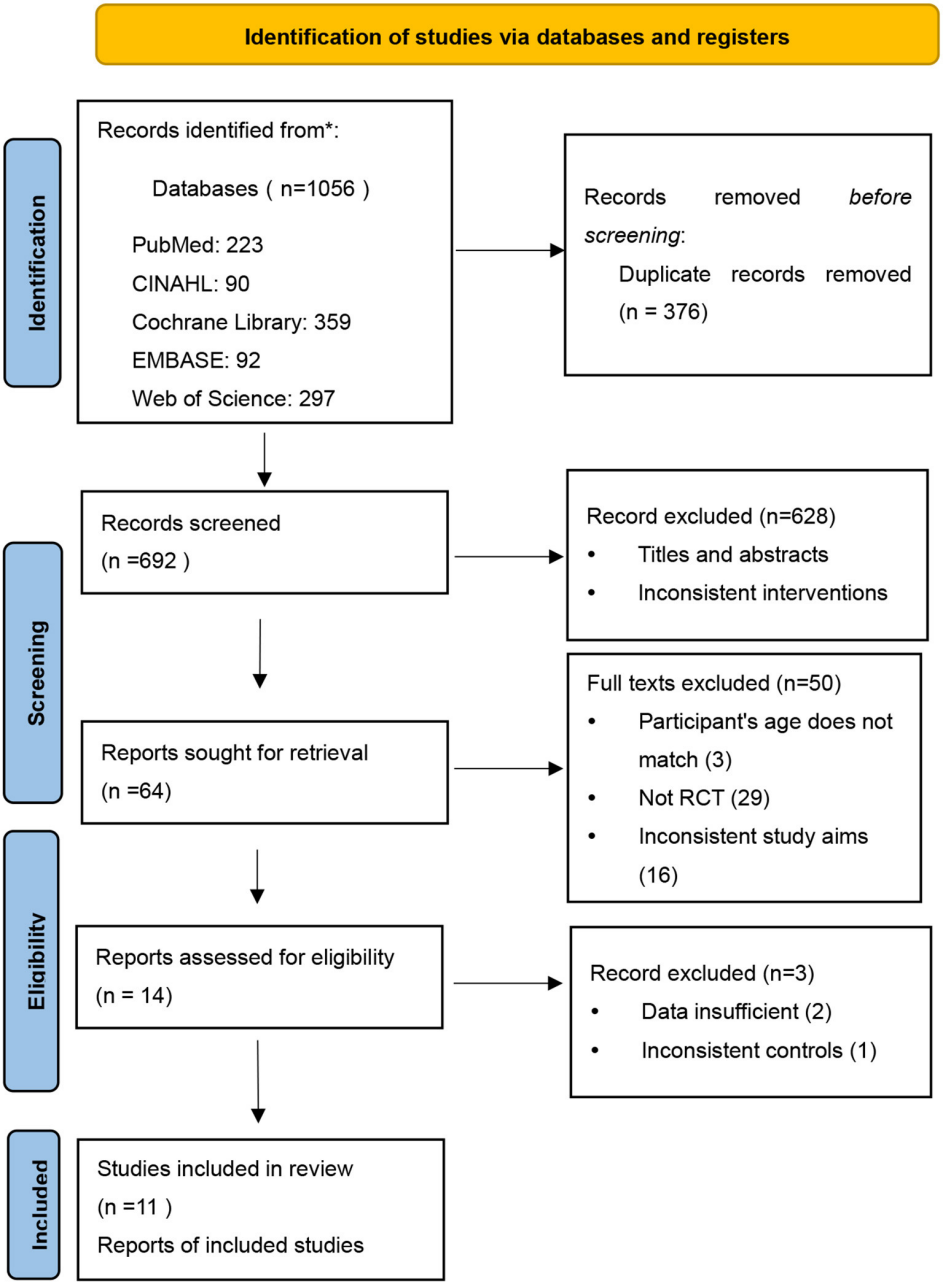
A flow diagram of the studies included in the review.

A total of 1,056 articles were retrieved, of which 64 met the study criteria for inclusion (Figure 1). After constructing the full text, we finally included 14 studies for the review (Table 1). Nevertheless, three studies did not have enough data to pool the estimates of children's compliance with spectacle use, but descriptive data from these studies were still included in the review.

**Table 1:** Characteristics of selected studies included in the systematic review (*N* = 14).

**NO**.	**Study**	**Country**	**Age (years)**	**Intervention**	**Control**	**Definition of compliance**	**Compliance measure**	**Follow-up years**	**Sample size**	**No. compliant (%)**
1	Ethan et al. (24)	America	None	Free spectacle and teacher incentive	Spectacles only at the end of the study	Wearing	Direct observation in the classroom	None	Treatment group: 121 Control group: 127	41 (33.88%)
2	Du et al. (19)	China	10.46 ± 1.08	Free spectacle	Spectacle voucher	Wearing	Unannounced visits	7	Treatment group: 1,055 Control group: 771	439 (41.6%)
3	Thapa et al. (16)	Nepal	13 ± 4	Free spectacle	Eye screening only	Wearing	Unannounced visits	6	Treatment group: 109 Control group: 145	62 (57%)
4	Holguin et al. (6)	Mexico	10.4 ± 2.6	Free spectacle	None	Wearing	Direct inspection	18	Treatment group: 493 Control group: None	66 (13.9%)
5	Yi et al. (17)	China	10.9	Free spectacle, education on their use, and teacher incentive	Spectacle prescriptions only	Wearing	Unannounced visits and children self-reported	6	Treatment group: 341 Control group: 352	233 (68.3%)
6	Guan et al. (20)	China	10.53	A voucher that can be redeemed for free spectacles	Spectacle prescriptions only	Wearing	unannounced visits	7	Treatment group: 971 Control group: 1,018	825 (85%)
7	Nie et al. (22)	China	13.56 ± 1.14	Free spectacles	Spectacles only at the end of the study	Wearing	Children self-reported	3	Treatment group: 476 Control group: 434	343 (72%)
8	Keay et al. (13)	China	14.1 ± 0.9	Free spectacles	None	Wearing	Unannounced visit	1	Treatment group: 415 Control group: NONE	193 (46.5%)
9	Wedner et al. (15)	Tanzania	14.4	Free spectacles	Spectacle prescriptions only	Wearing	Unannounced visit	3	Treatment group: 58 Control group: 50	27 (46.55%)
10	Wang et al. (18)	China	10.6 ± 1.0	A: Free spectacles B: Free spectacles + offer of $15 Upgrade spectacles C: Free spectacles + offer of $30 Upgrade spectacles	Spectacle prescriptions only	Spectacle purchase and wearing	Unannounced visits and children self-reported	6	Treatment group: 252M Control group: 250	80 (31.75%)
11	Ma et al. (5)	China	10.5 ± 1.1	Vouchers for free spectacles at a local facility, or free spectacles provided in class	Spectacle prescriptions only	Wearing	Unannounced direct examinations	8	Treatment group: 1,104 Control group: 1,033	453 (41.03%)
12	Ma et al. (21)	China	13.56	Free spectacles	Spectacle prescriptions only	Wearing	Unannounced visit	9	Treatment group: 476 Control group: 434	209 (43.91%)
13	Ma et al. (23)	China	10–12	Early referral to the vision center for refraction and free spectacles	Late referral for the identical intervention	Wearing	Children self-reported	6	Treatment group: 1,461 Control group: 1,252	806 (55.17%)
14	Ma et al. (14)	China	None	Free vision care and glasses	Spectacles only at the end of the study	Eyeglasses ownership and wearing	Children self-reported	3	Treatment group: 433 Control group: 516	347 (80.14%)

All of the studies included in this review were RCTs. All the data analyzed were based on previously published studies that received ethical approval and patient consent. Two studies were excluded from the meta-analysis because the data were insufficient (6, 13), and one study was excluded because the measures of the control group were inconsistent (14).

The 14 selected articles were conducted in five countries, and 67% were conducted in China. Sample sizes ranged from 108 to 2,713 (total *N* = 14,147 participants who were randomized, and *N* = 12,290 who contributed data to the synthesis). The overall compliance with spectacle use was 53.11% (*n* = 4,124/7,765). The age inclusion criteria ranged from 5 to 18 years (grades ranged from 1 to 8). The duration of follow-up visits ranged from 1 to 18 months. The differences in age and duration of follow-up may have contributed to the greater heterogeneity of the studies.

Children in the intervention group received free spectacles, and these children were considered compliant if they continued wearing spectacles at the end of the follow-up. Compliance was measured by experimenter observation or children's self-report. Of the included studies, nine assessed unannounced observed spectacle wear as their primary outcome (5, 13, 15–21), while three studies were conducted to collect children's self-reported wearing data as their primary outcome (14, 22, 23).

### 3.2. Spectacle compliance

**Figure 2:**
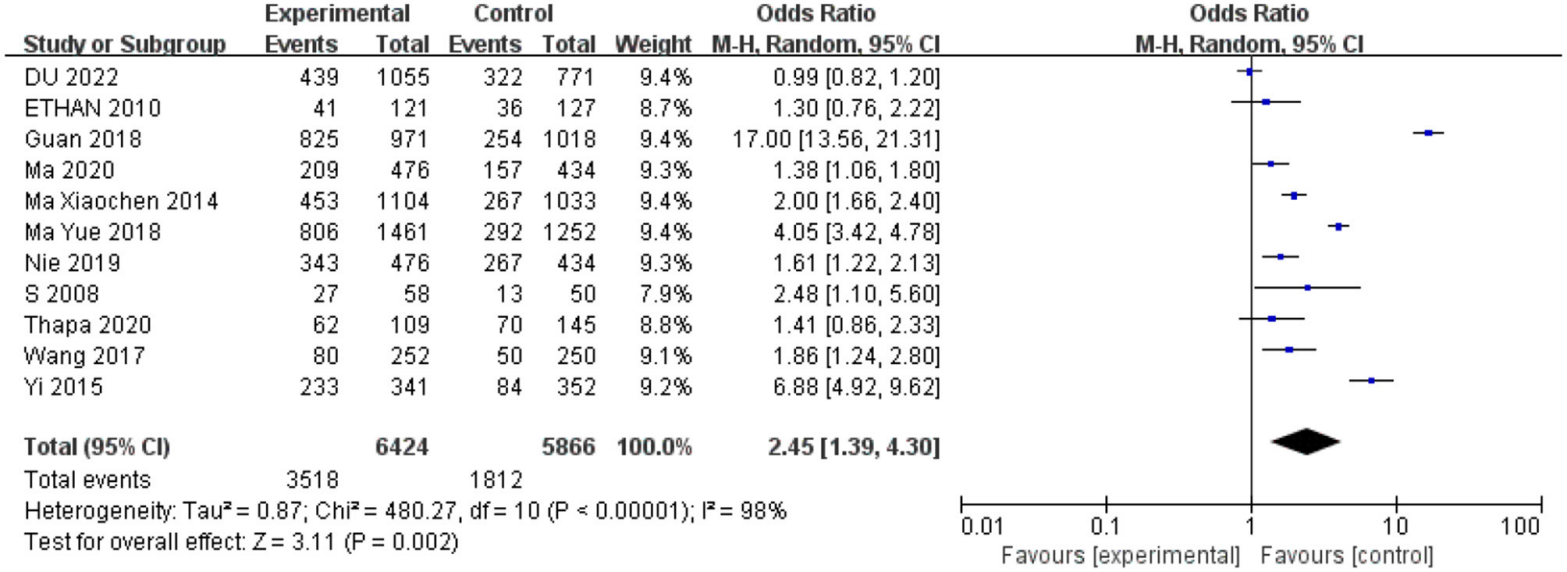
Random-effect meta-analyses of the spectacle-wear compliance (*N* = 11).

A total of 11 studies reported the number of children compliant with spectacle wear and were included in the meta-analysis. Since the heterogeneity was significant (*I*^2^ > 50%, *P* < 0.5), the random effects model was used for the analysis. The two methods of providing only a prescription (5, 10, 15, 22) or a prescription and a letter to the parents (5, 17, 18, 20, 21, 25) had lower compliance than the measure of providing free eyeglasses. There was statistical significance in the effect of the receipt of free spectacles on children's compliance with wearing spectacles (odds ratio = 2.45; 95%CI = 1.39–4.30; Figure 2).

### 3.3. Sensitivity analysis

**Figure 3:**
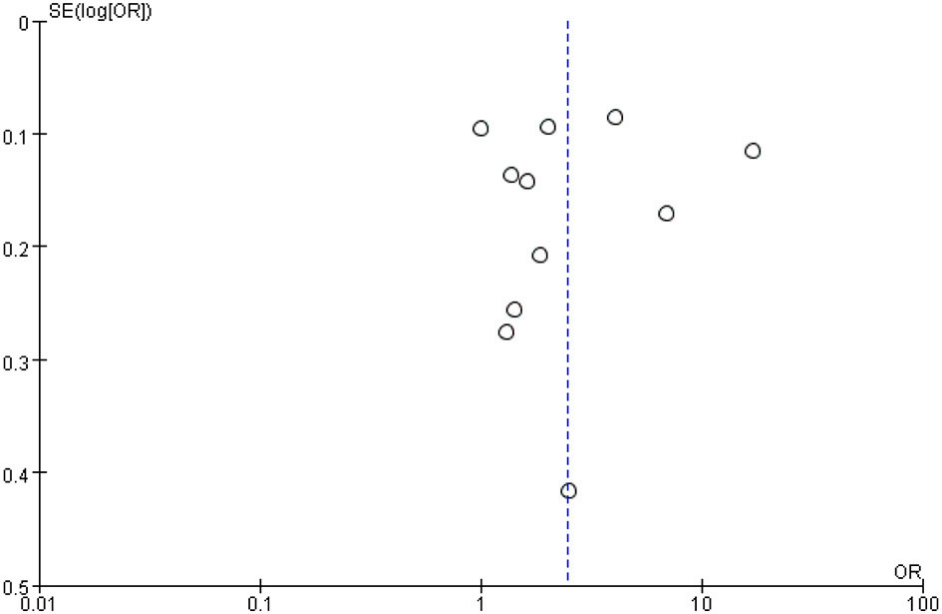
A funnel plot for the sensitivity analysis.

The results of the sensitivity analyses are presented in Figure 3. The odds ratio (OR) values were similar while excluding any study, and the *P*-values were all < 0.05. There was no significant difference in the *I*^2^ values while excluding any individual study. The Galbraith plot (Supplementary Figure 1) showed that there was significant heterogeneity among the studies. The heterogeneity could be attributed to the large age difference in the included samples and the different follow-up times.

### 3.4. Risk of bias

**Figure 4:**
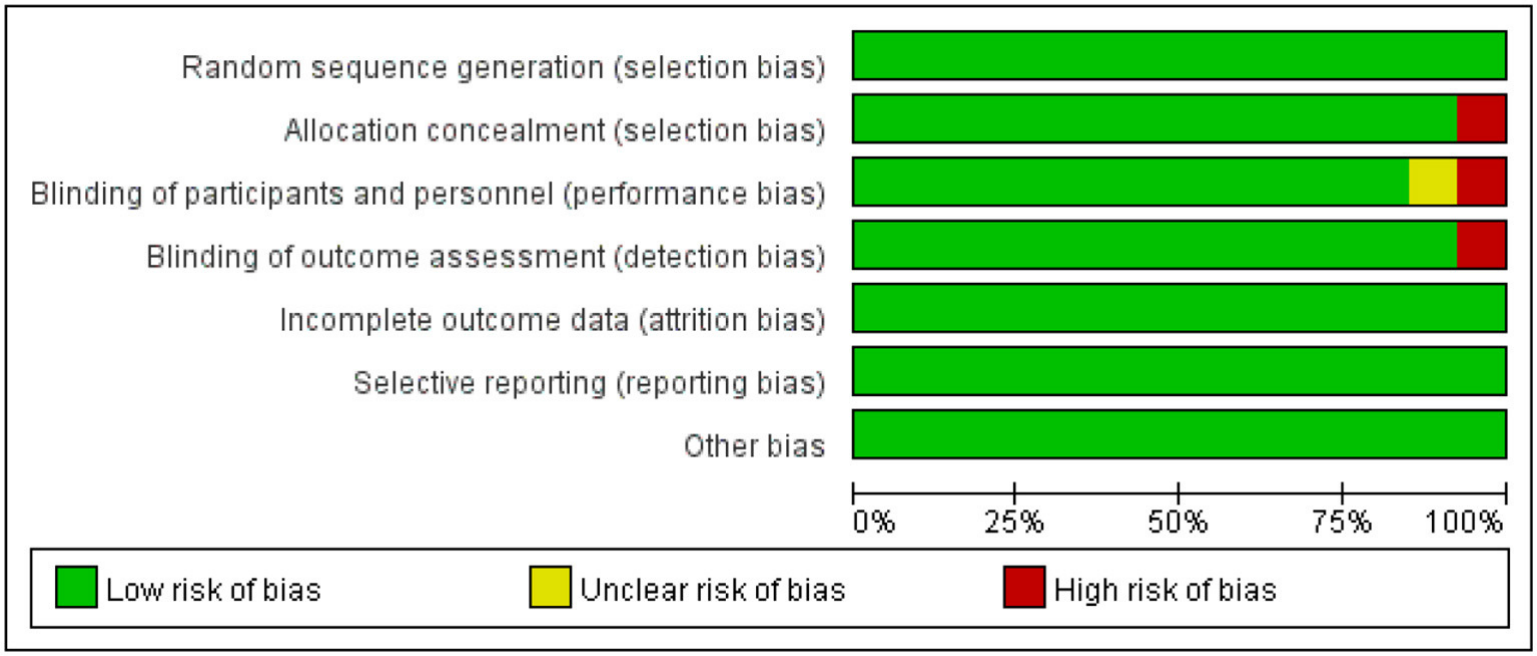
Risk of bias graph for included studies.

The risk of bias in the studies was assessed by one author using the Cochrane Risk of Bias Tool. Of the 11 articles, most (90%) reported a moderate to low risk of bias in Figure 4.

### 3.5. Subgroup analysis

**Figure 5:**
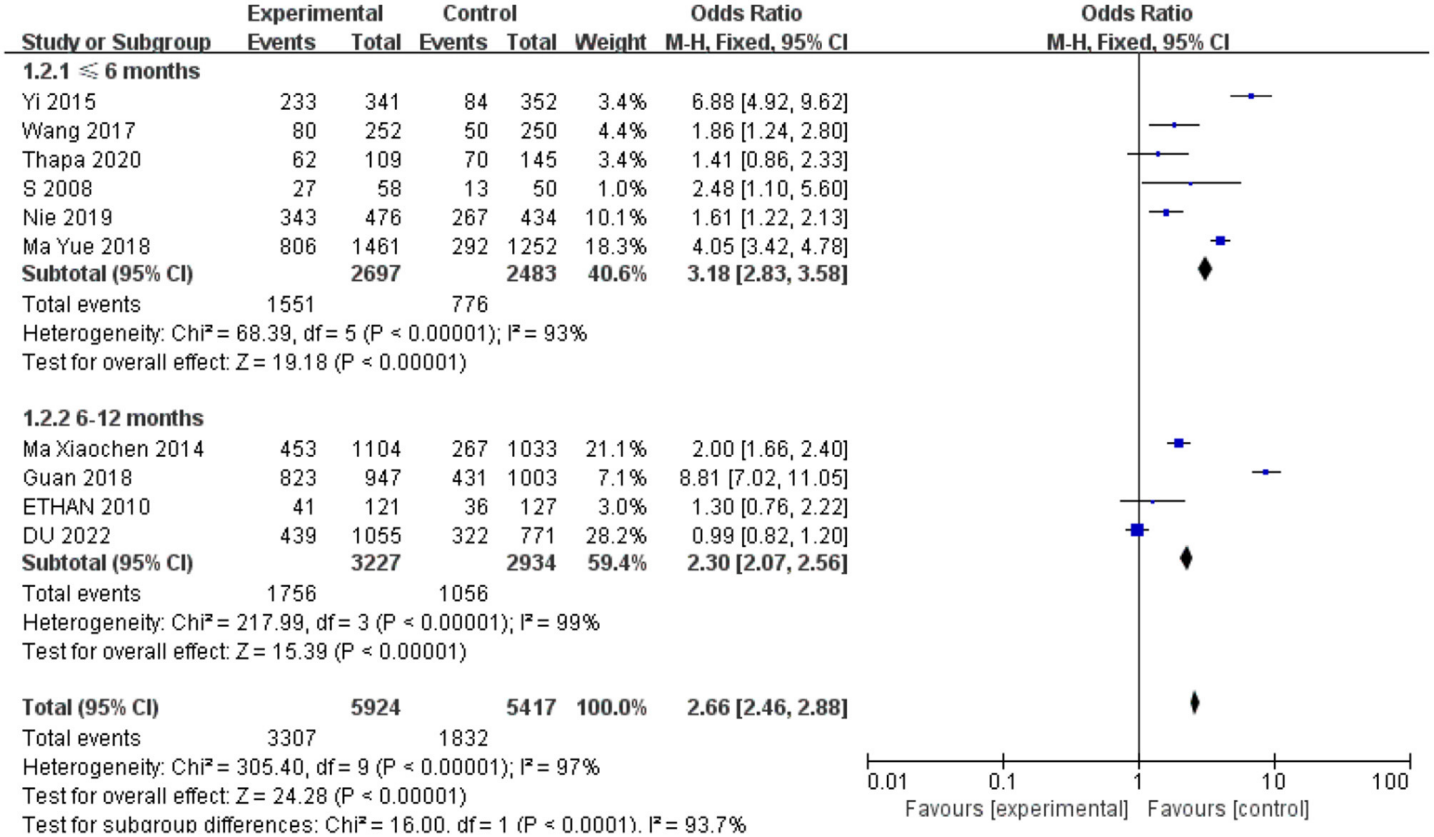
A subgroup analysis of included studies for follow-up time.

In the subgroup analysis, 6–12 months of follow-up time was associated with a significantly smaller OR value (OR = 2.30, 95% CI: 2.07–2.56) compared to < 6 months of follow-up time for studies (OR = 3.18, 95%CI: 2.83–3.58) evaluating associations between receipt of free spectacles and affected children's compliance with spectacle use in Figure 5.

**Figure 6:**
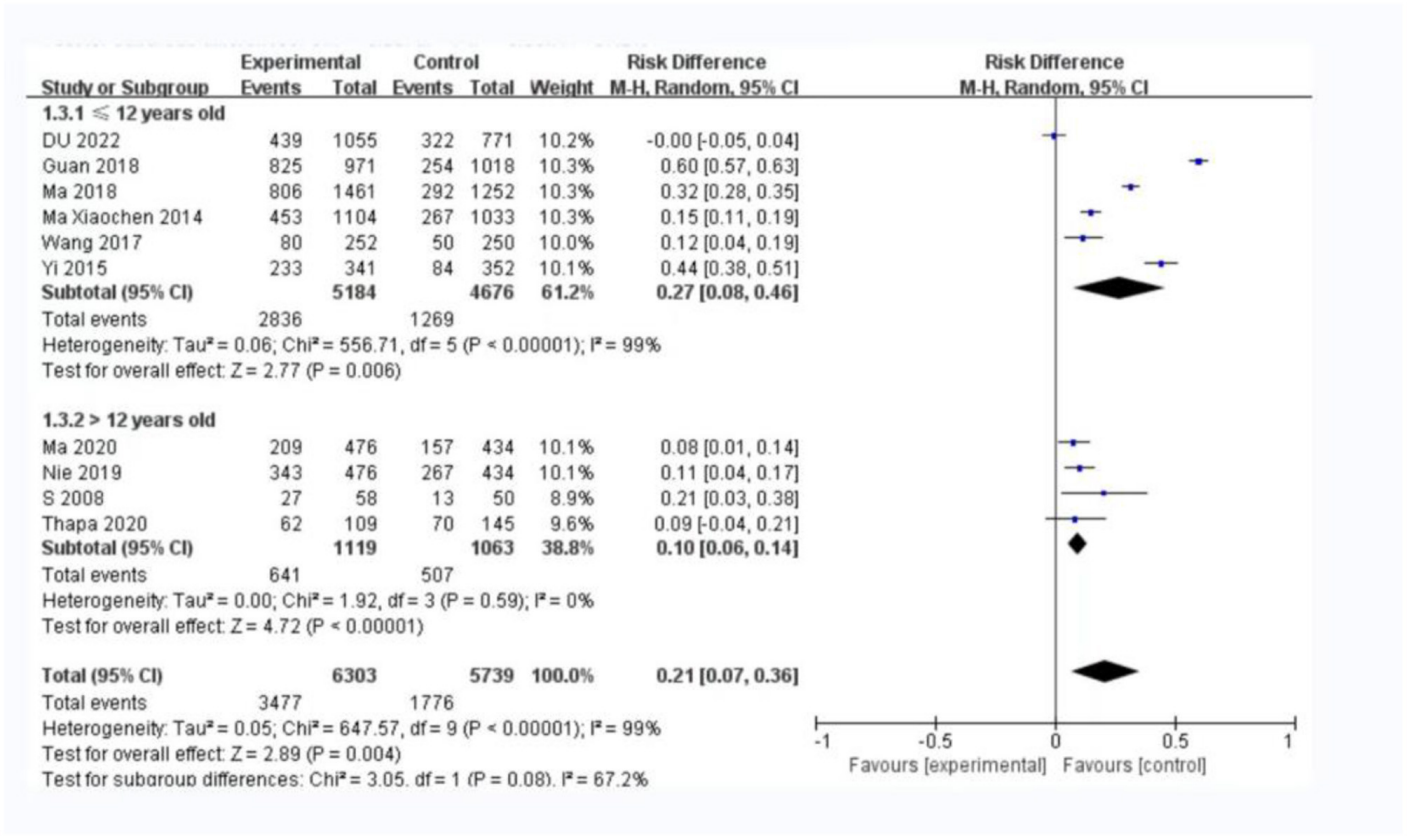
A subgroup analysis of the mean age of the participants.

In the subgroup analysis of participants' age, we found a smaller OR value (OR = 0.10, 95% CI: 0.06–0.14) in the Figure 6 in which the mean age of participants was over 12 years old compared with the subgroup in which their mean age was ≤ 12 years old (RR: 0.27, 95% CI: 0.08–0.46).

### 3.6. Factors associated with spectacle wear

Seven studies identified the factors contributing to children's use of spectacles at the end of follow-up after receiving free spectacles (6, 13, 17–20, 22). These included sociodemographic factors, the severity of the refractive error, and other factors in Table 2.

**Table 2:** Reasons for compliance with spectacle use.

**Study**	**Factors associated with spectacle wear**
Holguin et al. (6)	Older (OR: 1.19 per year of age; 95% CI: 1.05–1.33), rural (OR: 10.6; 95% CI: 5.35–21.0) children, and those with myopia (OR: 3.97; 95% CI: 1.98–7.94) and hyperopia (OR: 3.63; 95% CI: 1.02–12.9)
Du et al. (19)	Older (RR: 1.56, 95%CI: 1.12–2.19), severity of RE < −2.00 (RR: 3.68; 95% CI: 2.23–6.07), wearing spectacles before baseline (RR: 3.91; 95% CI: 2.53–6.04), friends wear spectacles (RR: 1.87; 95% CI: 1.32–2.63)
Keay et al. (13)	Female (OR: 1.72; 95% CI: 1.10–2.68), low income (OR: 1.78; 95% CI: 1.32–2.39), less trouble with appearance (OR: 2.04; 95% CI: 1.25–3.36)
Guan et al. (20)	Left-behind children
Wang et al. (18)	Baseline wearing (RR: 3.01, 95% CI: 2.32–3.89), uncorrected visual acuity (children with better visual acuity were less likely to wear spectacles: RR: 0.24; 95% CI: 0.11–0.50, *P* < 0.001)
Yi et al. (17)	Membership in the treatment group (OR: 11.5, 95% CI: 5.91–22.5), baseline wearing (OR: 12.2; 95% CI: 5.63–26.4), VA < 6/18 both eyes (OR: 1.70; 95% CI: 1.14–2.53), and at least one parent wears spectacles (OR: 1.90; 95% CI: 1.14–3.18)
Ma et al. (23)	Membership in the treatment group (OR: 11.89, 95% CI: 6.77–20.89), baseline ownership (OR: 31.82; 95% CI: 10.24–98.91), baseline mathematics score (OR: 1.19; 95% CI: 1.08–1.31)
Thapa et al. (16)	Lack of awareness of the need for distance glasses by the children's carers

Sociodemographic factors include children's age (or grade), sex, family income, urban vs. rural residence, and whether they were left behind, but this was not consistent with the findings of the studies. Holguin et al. (6) reported that older and urban-dwelling children were more likely not to wear spectacles, while Du et al. (19) concluded that older children were more compliant with free spectacles. Keay et al. reported that children from low-income families were more likely to wear spectacles. In the study by Guan et al. (20), left-behind children were more likely to wear spectacles than children who were not left behind.

Other factors that influenced compliance were whether children were in their seats to see the blackboard clearly and the attitude of their parents. Children who could not see the blackboard had higher compliance (19). In two studies including a teacher motivation incentive in addition to free spectacles in the treatment group (17, 23), the reported rate of wearing spectacles was 55.2 and 68.3%. Children's baseline spectacle wear largely determined whether they wore them during follow-up (17, 18). Children with higher levels of refractive error were more likely to wear spectacles than children with lower refractive errors (6, 17–19). Children might be concerned about their appearance and being teased by their classmates (26). The appearance of spectacles was a factor in the acceptance of spectacles by children (13). However, in studies where children were allowed to choose their own frames, fewer than half of the children were found to wear spectacles (13, 24).

## 4. Discussion

This systematic review included 14 RCTs that evaluated interventions that demonstrated that providing free spectacles can increase the use of children's spectacles. Some of these RCTs reported that fewer than half of the children wore their spectacles at the follow-up time, even when they were provided for free. However, it was more effective to provide free spectacles than to provide only a prescription or a prescription and a letter to the parents.

A combination of interventions may have increased children's compliance. The reasons reported by Yi et al. (17) for the increase in compliance were receipt of free spectacles combined with education on their use and a teacher incentive. A similar study on strengthening health education intervention in India showed that, in this experiment, education intervention alone did not improve the compliance of glasses wearers (27). In the study by Ma et al. (23), teachers were trained in VA screening and screened the children during the intervention in the treatment group, which may have encouraged children to wear spectacles. The provision of free spectacles may have brought additional benefits to children. Existing evidence shows that free spectacles can improve academic performance (5, 14, 22). The results of the subgroup analysis showed lower ORs at the longer follow-up of 6–12 months. Our study's results may provide important insights for refractive services in children. For example, the results suggest that incorporating education and teacher incentives into long-term follow-up programs could encourage consistent spectacle use among children. At short-term follow-up, children may stop wearing spectacles because of the associated discomfort. Wearing spectacles during long-term follow-up may help children develop habits that allow them to benefit from refractive correction.

As the number and proportion of children with refractive errors are increasing worldwide, concerns have been raised about the increasing cost of eye care programs (8–10). Considering the fact that spectacle wear compliance was low among school children in some screening programs, it is necessary to adjust the strategies to improve compliance. A randomized clinical trial conducted in India found that using readymade spectacles can be a viable option for delivering refractive services in settings with a high level of need, limited resources, and low access to refractive services (28). The results of the study indicated that the cost of spectacles may be a barrier for children in low- and middle-income areas to access. Moreover, the reasons for non-compliance may include poor literacy, misconceptions, and lack of eye health knowledge among parents (29). Because economic considerations are important in low- and middle-income countries, the provision of low-cost or free spectacles can improve access (30).

To our knowledge, this systematic review is the first to focus on the provision of free spectacles to promote spectacle-wearing compliance in children. However, this study has several main limitations. First, there was a high degree of heterogeneity between the studies, reflecting methodological differences. The included studies differed considerably in the definition and measurement of compliance. Most studies on spectacle wear compliance used observed wear or relied on self-report. Observed wear is influenced by the degree of refractive error but cannot be fully explained (7). Self-reported spectacle wear may have resulted in overestimating the results of the study. Second, the majority of the studies were from the Asia region, which may reduce the representativeness of the results. Third, not all of the included studies were specifically designed to investigate children's compliance with free spectacles.

## 5. Conclusions

In conclusion, our systematic review and meta-analysis showed that providing free spectacles with educational interventions significantly improves children's compliance with spectacle wear. These findings highlight the importance of incorporating educational interventions and other health promotion strategies in policies aimed at increasing spectacle compliance. However, further research is needed to better understand the reasons for low compliance and to develop more effective measures for improving the acceptance of refractive services and eyewear use.

## Data availability statement

The original contributions presented in the study are included in the article/Supplementary material, further inquiries can be directed to the corresponding author.

## Author contributions

LW: software, formal analysis, data curation, and writing—original draft. JF: data visualization and writing—original draft. MZ: conception and design of study. All authors contributed to the article and approved the submitted version.
