# Supplementary figures and images for: Implementing interventions to promote spectacle wearing among children with refractive errors: A systematic review and meta-analysis
Source: Front Public Health. 2023 Mar 10;11:1053206. doi: 10.3389/fpubh.2023.1053206 (PMC10036364; doi:10.3389/fpubh.2023.1053206)

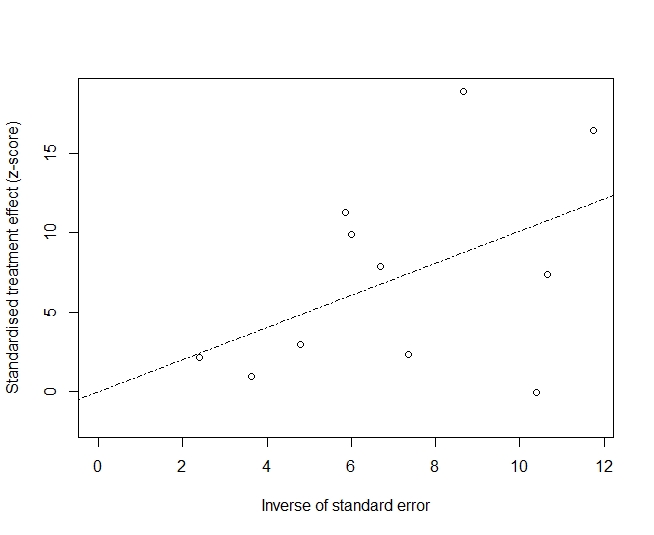

Supplement: Supplementary file 1 [file Image_1.jpg]
